# An Insight on the Pathways Involved in Crizotinib and Sunitinib Induced Hepatotoxicity in HepG2 Cells and Animal Model

**DOI:** 10.3389/fonc.2022.749954

**Published:** 2022-01-28

**Authors:** Lin Guo, Tingli Tang, Dongmei Fang, Hui Gong, Bikui Zhang, Yueyin Zhou, Leiyi Zhang, Miao Yan

**Affiliations:** ^1^ Department of Pharmacy, The Second Xiangya Hospital, Central South University, Changsha, China; ^2^ School of Pharmaceutical Sciences, Sun Yat-Sen University, Guangzhou, China; ^3^ Orthodontic Department of Xiangya Stomatology Hospital, Central South University, Changsha, China; ^4^ Department of General Surgery, The Second Xiangya Hospital, Central South University, Changsha, China

**Keywords:** crizotinib, sunitinib, hepatotoxicity, Keap1/Nrf2, apoptosis, liver mitochondrial injury

## Abstract

Both crizotinib and sunitinib, novel orally-active multikinase inhibitors, exhibit antitumor activity and extend the survival of patients with a malignant tumor. However, some patients may suffer liver injury that can further limit the clinical use of these drugs, however the mechanisms underlying hepatotoxicity are still to be elucidated. Thus, our study was designed to use HepG2 cells *in vitro* and the ICR mice model *in vivo* to investigate the mechanisms of hepatotoxicity induced by crizotinib and sunitinib. Male ICR mice were treated orally with crizotinib (70 mg/kg/day) or sunitinib (7.5 mg/kg/day) for four weeks. The results demonstrated that crizotinib and sunitinib caused cytotoxicity in HepG2 cells and chronic liver injury in mice, which were associated with oxidative stress, apoptosis and/or necrosis. Crizotinib- and sunitinib-induced oxidative stress was accompanied by increasing reactive oxygen species and malondialdehyde levels and decreasing the activity of superoxide dismutase and glutathione peroxidase. Notably, the activation of the Kelch-like ECH-associated protein-1/Nuclear factor erythroid-2 related factor 2 signaling pathway was involved in the process of oxidative stress, and partially protected against oxidative stress. Crizotinib and sunitinib induced apoptosis *via* the mitochondrial pathway, which was characterized by decreasing Bcl2/Bax ratio to dissipate the mitochondrial membrane potential, and increasing apoptotic markers levels. Moreover, the pan-caspase inhibitor Z-VAD-FMK improved the cell viability and alleviated liver damage, which further indicated the presence of apoptosis. Taken together, this study demonstrated that crizotinib- and sunitinib-caused oxidative stress and apoptosis finally impaired hepatic function, which was strongly supported by the histopathological lesions and markedly increased levels of serum alanine aminotransferase, alkaline phosphatase and lactate dehydrogenase.

## Introduction

Crizotinib, an oral inhibitor of anaplastic lymphoma kinase, MET proto-oncogene, and c-ros oncogene 1 tyrosine kinases, was approved by the U.S. Food and Drug Administration (FDA) in 2011 for non-small cell lung cancer (1). Although crizotinib has been documented to improve survival in cancer patients, it can cause severe adverse effects, including pulmonary toxicity (2), acute and fulminant hepatitis (3). In clinical trials, the frequency of elevated serum transaminases in patients treated with crizotinib was 10-38% for all grades, 16% for grade 3 to grade 4 and nearly 0.1% for fatal hepatotoxicity (4). Recently, two clinical cases reported that patients treated with crizotinib presented with fatal liver failure despite the discontinuation of crizotinib and intensive supportive therapy (5, 6).

As another oral multitargeted inhibitor of platelet-derived growth factor receptors, vascular endothelial growth factor receptor and c-Kit tyrosine kinases, sunitinib was approved by FDA in 2006 for patients with metastatic renal-cell carcinoma, imatinib-resistant gastrointestinal stromal tumors and pancreatic neuroendocrine tumors (7). Nevertheless, sunitinib showed some potentially severe adverse reactions including cardiac dysfunction and potentially life-threatening hepatotoxicity (8, 9). Sunitinib-induced liver failure has been reported in many clinical cases (10). In clinical trials, 2-5% of patients treated with sunitinib developed grade 3 and grade 4 elevated aminotransferase (11) and hepatic failure happened in 0.3% of patients (12). The US FDA requested a black box warning of hepatotoxicity for the use of sunitinib pending warnings of fatal liver damage reports in 2010 (4). Hepatotoxicity has limited the clinical application of crizotinib and sunitinib. Therefore, there is an urgent need to further explore the molecular mechanisms and pathways associated with crizotinib- and sunitinib-induced hepatotoxicity for clinical medication guidance and hepatotoxicity avoidance. Recently, researchers have reported that crizotinib did not significantly affect mitochondrial function in isolated rat liver mitochondria (13) and HepG2 cells (14) at concentrations of 20- to 100-fold peak blood levels. However, some *in vitro* studies suggested that crizotinib induced ATP depletion, caspase activation in primary rat and human hepatocytes (15), and reactive oxygen species (ROS) generation in HL7702 cells (16). Similarly, Zhang and his colleagues reported that sunitinib showed no effects on intact mitochondria or submitochondrial particles even at the highest concentrations tested in isolated rat liver mitochondria (13). Nevertheless, recent research showed that sunitinib generated toxic metabolites causing mitochondrial toxicity in mice (17, 18), and apoptosis was induced in HepG2 cells and HepaRG cells (19). The results of the previous studies appear to be incompatible or contradictory in different cell lines and animal models. Thus, it is important to investigate whether oxidative damage and mitochondrial-related apoptosis are involved in crizotinib- and sunitinib-induced hepatotoxicity. Therefore, the present study was conducted using HepG2 cells as an *in vitro* model and ICR mice as an *in vivo* model to explore potential mechanisms associated with crizotinib- and sunitinib-induced hepatotoxicity. Our results confirmed that crizotinib and sunitinib treatment induced liver toxicity, which manifested in terms of elevated liver enzymes, elevated oxidative stress, and mitochondrial dysfunction, which subsequently lead to hepatocyte apoptosis. Importantly, we were the first to find that the Kelch-like ECH-associated protein-1 (Keap1)/Nuclear factor erythroid-2 related factor 2 (Nrf2) signaling pathway was involved in the process of crizotinib- and sunitinib induced oxidative stress. Our findings indicate that the activation of the Keap-Nrf2 pathway may participate in the elimination of ROS to alleviate oxidative injury.

## Materials And Methods

### Drugs and Reagents

Crizotinib (purity≥98%) and sunitinib (purity≥99%) were obtained from Huateng pharmaceuticals-company (Hunan, China). DMEM medium and phosphate-buffered saline (PBS) were obtained from Gibco (Grand Island, NY, USA). Fetal bovine serum (FBS) was obtained from Biological Industries (Israel). Dimethyl sulfoxide (DMSO) and 3-(4, 5-dimethylthiazol-2-yl)-2, 5-diphenyltetrazolium bromide (MTT) were obtained from Sigma-Aldrich (St. Louis, MO, USA). Trypsin, penicillin, and streptomycin were obtained from Hyclone (Logan, USA). The primary antibodies used were anti-Nrf2 (sc-722, Santa Cruz), anti-Keap1 (af5266, Affinity), anti-cleaved caspase3 (af7022, Affinity), anti-Bcl2 (ab692, Abcam), anti-Bax (ab32503, Abcam), anti-Histone H3 (af0863, Affinity), and anti-β-actin (ac006, ABclonal).

### HepG2 Cell Culture

HepG2 cells were cultured in DMEM medium supplemented with 10% FBS and 1% streptomycin and penicillin. The cells were maintained in a water-jacket CO_2_ incubator at 37°C with 5% CO_2_. In all experiments, the cells were inoculated with an appropriate density according to the experimental design and cultured for 24 h before the treatment.

### Animal Treatment and Drug Administration

ICR male mice (body weight of 18-22 g) were purchased from Hunan Slack Jingda Experimental Animal Co., Ltd. (Hunan, China). The mice were acclimatized for one week and were maintained under a standard conditioned environment. Water and normal chow were given ad libitum. Animal care was following institutional guidelines. The study was approved by the Institutional Animal Care and Use Committee of Central South University (Hunan, China). The mice were randomly divided into vehicle-treated group (control, n=8), crizotinib-treated group (n=8, 35 mg/kg, twice daily) and sunitinib-treated group (n=8, 7.5 mg/kg/day, once daily). The mice received either 0.5% (w/v) carboxymethyl cellulose sodium once daily, crizotinib twice daily or sunitinib once daily *via* intragastric administration for 4 weeks consecutively. After 24 h of the last treatment, the animals were euthanized, blood samples were collected and livers were surgically excised and collected in 10% phosphate-buffered formalin for further determination.

### Cytotoxicity Assay

HepG2 cells were seeded (5×10^3^ cells/well) in 96-well plates, with 200 μL media per well. Cells were exposed to different concentrations of crizotinib (0, 5, 10, 15, 20, 30, 40 μM) or sunitinib (0, 3.2, 6.6, 13.1, 19.6, 26.1, 39.2, 52.2 μM) for 12, 24, and 48 h. Cells were incubated with fresh MTT solution (100 μL/well; stock 5 mg/mL in PBS) for 3-4 h. After the crystal dissolved, the plates were read on an automated microplate spectrophotometer (Thermo Multiskan Spectrum, Thermo Electron Corporation, USA) and absorbance at 570 nm was measured.

### Hepatotoxicity Assessments

After crizotinib and sunitinib treatment of HepG2 cells, the supernatant was collected and the biochemical parameters alanine aminotransferase (ALT), aspartic acid transferase (AST), alkaline phosphatase (ALP) and lactate dehydrogenase (LDH) were measured by the full-automatic clinical analyzer in the laboratory of the second Xiangya hospital (7600, HITACHI Ltd., Tokyo, Japan).

Liver samples of the mice were fixed in 10% phosphate-buffered formalin and embedded in paraffin. In brief, the liver tissue was embedded in paraffin, then deparaffinized with xylene, stained with hematoxylin and eosin, then dehydrated and sealed, and finally evaluated for damage under light microscopy.

### Apoptosis Determined by Annexin V-FITC and TUNEL Assay

Apoptosis was detected through flow cytometry using FITC Annexin V Apoptosis Detection Kit (Bestbio, Shanghai, China). Drug-treated cells (culture in the incubator for 24 h) were digested by trypsin without EDTA, centrifuged, and resuspended with PBS for 3 times strictly. The fluorescence maker was added and cells were incubated in a dark place at 2-8°C for 15 min, followed by sample loading and detection through flow cytometry. All samples were analyzed within 1 h to ensure the effect.

Terminal deoxynucleotidyl transferase-mediated dUTP nick end labeling (TUNEL) assay was conducted with the TUNEL kit according to the manufacturer’s instructions. In brief, the liver tissue was embedded in paraffin, then deparaffinized with xylene, stained with TUNEL reaction mixture, then stained by DAPI staining and anti-fluorescence quenching were performed. Finally, the obtained slices were observed and photographed at a suitable high magnification, with the apoptotic cells appearing green and the nuclei appearing in blue.

### Accumulation of ROS

The level of ROS was determined using the fluorescent probe DCFH-DA (Beyotime Biotechnology, Shanghai, China). HepG2 cells (3.5×10^5^ cells/well) were treated with different concentrations of crizotinib (0, 8, 15, 20 μM) or sunitinib (0, 5, 9, 14 μM) for 24 h. After DCFH-DA was added at a final concentration of 10.0 μM to the culture medium, the hepatocytes in 24-wells were incubated at 37°C for an additional 20-30 min, and then washed with PBS, and measured immediately by fluorescence microscope (Thermo Electron Corporation, USA). Increased green fluorescence intensity was used to quantify intracellular ROS production.

### Measurement of Glutathione Peroxidase (GPx), Superoxide Dismutase (SOD) and Malondialdehyde (MDA)

The extent of oxidative stress was estimated in liver homogenates by measuring activities of GPx, SOD and MDA using commercial kits (Jiancheng Bioengineering Institute, Nanjing, China) according to the manufacturer’s instructions. GPx is an important selenoprotein that reduces hydroperoxides as well as hydrogen peroxide (H_2_O_2_) while oxidizing glutathione, which can protect the structure and function of the cell membrane (20, 21). Briefly, GPx can promote the reaction of H_2_O_2_ with reduced glutathione (GSH) to produce H_2_O and glutathione oxidized. The activity of GPx was measured by spectrophotometer assay at 412 nm from the oxidation of GSH in the presence of H_2_O_2_ used as substrate.

The activity of SOD was determined by the xanthine oxidase (hydroxylamine) method. This redox produced superoxide which oxidizes hydroxylamine to nitrite by reacting with the reagent producing a purple-red dye. The absorbance of the color which was inversely proportional to the SOD activity was determined by a spectrophotometer at 550 nm (22).

The production of MDA was assessed with the thiobarbituric acid reactive substances method (TBA). TBA was added to each sample tube and vortexed. The reaction mixture was incubated at 95°C for 60 min. After cooling, the pink pigment was read spectrophotometrically at 532 nm (22).

### Mitochondrial Membrane Potential (MMP)

Mitochondrial membrane potential assay kit with JC-1 (Beyotime Biotechnology, Shanghai, China) is a fast and sensitive assay kit that uses JC-1 as a cationic dye to detect membrane potential changes in cells, tissues or purified mitochondria, which can be used for early detection of apoptosis. After the liver tissue was digested, cell precipitation was collected, then fluorescence probe was loaded and cells were incubated at 37°C for 20 min, mixed well every 3-5 min, and washed with dyeing buffer (1×) at 4°C and centrifuged three times, finally detected by flow cytometry.

### Western Blotting

The HepG2 cell and animal liver protein samples were extracted with enhanced RIPA lysate (Boster, Hubei, China), the cytoplasmic and nuclear proteins were prepared with the subcellular structure cell nucleus and cytoplasmic protein extraction kit (Boster, Hubei, China) according to the manufacturer’s instruction. The protein concentration of whole-cell lysates was determined using the BCA method (Boster, Hubei, China). Protein lysates (15-30 μg) were loaded on 8-12% SDS-PAGE gels, separated electrophoretically and transferred to the PVDF membrane. Subsequently, the membranes were incubated in a blocking solution at room temperature for 1 h. After blocking, membranes were separately incubated at 4°C on a rocker with primary antibodies specific to the protein of interest; these were rabbit anti-Keap1 antibody (1:1000), anti-cleaved caspase3 antibody (1:1000), anti-Bax antibody (1:5000), anti-Histone H3 antibody (1:1000), anti-β-actin antibody (1:500-1:2000), mouse anti-Nrf2 antibody (1:800), and anti-Bcl2 antibody (1:500). Subsequently, the membranes were incubated with a suitable HRP-conjugated secondary antibody (Proteintech, USA) for 1h, and then signal detection was conducted with an ECL kit (Boster, Hubei, China) according to the manufacturer’s protocol.

### Statistical Analysis

The data were presented as the means ± standard derivation (SD). The significance of differences between groups was determined with the one-way analysis of variance (ANOVA) and SPSS 20.0 software (SPSS Inc., Chicago, IL, USA), and comparison between two groups was done with an independent sample t-test. Figures were drawn with GraphPad Prism 6 (GraphPad Software, La Jolla, CA, USA).

## Results

### Crizotinib and Sunitinib Induced Hepatotoxicity

**Figure 1:**
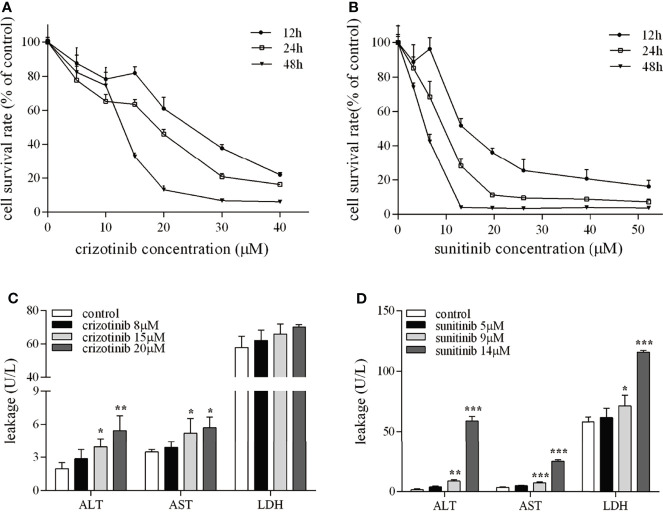
The hepatotoxicity of crizotinib and sunitinib *in vitro*. **(A, B)** Cytotoxicity of crizotinib or sunitinib alone at the various concentration for 12, 24 and 48 h in HepG2 cells (n=5-6). **(C, D)** ALT, AST and LDH levels in the supernatant following HepG2 cell treatment with crizotinib or sunitinib at different concentrations for 24 h (n = 3). *P < 0.05, **P < 0.01 or ***P < 0.001 (the crizotinib or sunitinib alone vs. control). ALT, alanine aminotransferase; AST, aspartic acid transferase; LDH, lactate dehydrogenase.

The results showed that HepG2 cell viability was reduced in a concentration- and time-dependence manner (**Figures 1A, B** **)**. When cells were treated for 24 h, crizotinib 15 μM and sunitinib 9 μM were used in subsequent experiments. The levels of ALT, AST, and LDH are sensitive markers of hepatocyte damage. **Figure 1C** showed that ALT and AST levels increased significantly in the supernatant from treated HepG2 cells at a concentration of 15μM and 20 μM, but LDH levels were not significantly altered in the crizotinib treatment compared to vehicle. According to **Figure 1D**, sunitinib treatment significantly elevated the levels of ALT, AST, and LDH compared to vehicle.

**Figure 2:**
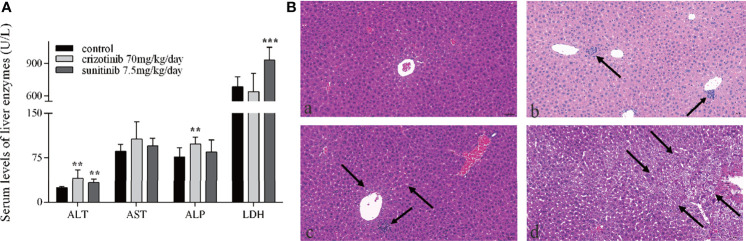
The hepatotoxicity of crizotinib and sunitinib *in vivo*. **(A)** Blood levels of different liver enzymes in male ICR mice after the administration of crizotinib or sunitinib treatment (n = 8). **(B)** Histopathological analysis of liver micro-tissues from animals in the different experimental groups. Representative images from: (a) control group; (b) crizotinib-treated animals, 70 mg/kg/day; (c, d) sunitinib-treated animals, 7.5 mg/kg/day. Magnification of photomicrographs at 20x. **P < 0.01 or ***P < 0.001 (the crizotinib or sunitinib alone vs. control). ALT, alanine aminotransferase; AST, aspartic acid transferase; ALP, alkaline phosphatase; LDH, lactate dehydrogenase.

Serum levels of the hepatic enzymes ALT and ALP were significantly elevated in crizotinib-treated group, while the levels of ALT and LDH were significantly elevated in animals treated with sunitinib compared to the control group (**Figure 2A**). Also, histopathological analysis of liver sections from the crizotinib group (**Figure 2B-b**) showed small pockets of inflammatory cells infiltrate around the hepatic lobules and the central veins, compared with those of the control group (**Figure 2B-a**). More hepatocyte edema, cytoplasm loose light dye, and a small amount of hepatocyte edema to balloon-like degeneration, cell swelling, cytoplasmic cavitation (**Figure 2B-c**), and a small amount of focal lymphocyte infiltration (**Figure 2B-d**) were seen in the sunitinib group, but not in the control group. These findings support drug-induced liver injury for animals treated with crizotinib and sunitinib *in vivo*.

### Hepatotoxicity Induced by Crizotinib and Sunitinib Is Mediated by Cell Apoptosis and Necrosis

**Figure 3:**
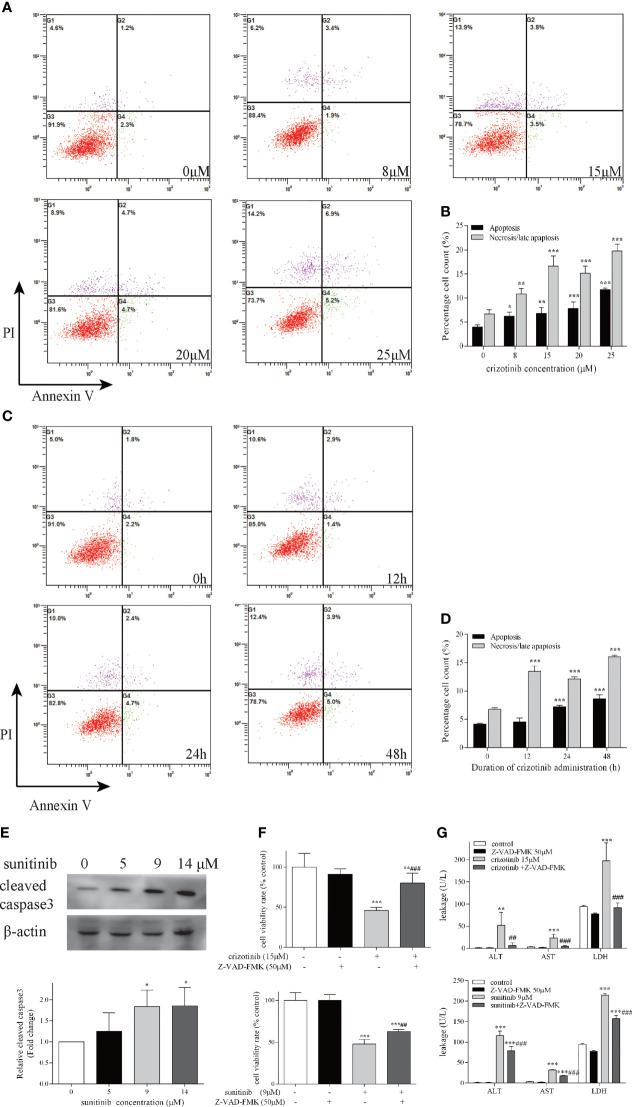
Crizotinib and sunitinib induced apoptosis in HepG2 cells. Following treatment of cells with increasing doses of crizotinib for 24 h **(A)** and increasing administration time of crizotinib 15 µM **(C)**, cell apoptosis was measured by Annexin V−FITC/PI double staining assay. In the flow cytometry plot, live, early apoptotic, late apoptotic and necrotic cells were shown in the lower left, lower right, upper right and upper left quadrants, respectively. **(B)** Quantification of experiments shown in **(A)**. **(D)** Quantification of experiments shown in **(C)**. **(E)** Western blot analysis for the level of cleaved caspase 3 after sunitinib exposure for 24 h (n=3). **(F)** The cell survival rate for HepG2 cells after treatment with crizotinib or sunitinib with or without Z-VAD-FMK. Cell viability was measured by the MTT assay, and **(G)** The levels of ALT, AST, and LDH in the supernatant of HepG2 cells treated with crizotinib or sunitinib with or without Z-VAD-FMK (n=3). In these experiments, cells were pretreated with Z-VAD-FMK 50 μM for 24 h before crizotinib (15 μM) or sunitinib (9 μM) treatment. *P < 0.05, **P < 0.01 or ***P < 0.001 (the crizotinib or sunitinib alone vs. control). ^##^P < 0.01 ^###^P < 0.001 (the crizotinib or sunitinib alone vs. the crizotinib or sunitinib pretreated with Z-VAD-FMK). ALT, alanine aminotransferase; AST, aspartic acid transferase; LDH, lactate dehydrogenase.

As shown in **Figure 3A**, an upward tendency pattern was apparent, when the HepG2 cells were treated with different concentrations of crizotinib (0, 8, 15, 20, 25 μM) for 24 h, which supported the hypothesis that crizotinib induced hepatocyte apoptosis and/or necrotic. Subsequently, a time-dependent increase was observed, and HepG2 cells treated for 24 h and 48 h with crizotinib showed greater apoptosis and/or necrosis (**Figure 3C**). The percentage of cells undergoing apoptosis and/or necrosis in crizotinib-treated hepatocytes increased dramatically compared with non-treated cells (**Figures 3B, D**). Activation of caspase 3 is the most critical apoptotic executive event in apoptosis. Sunitinib was associated with a significant concentration-dependent increase in cleaved caspase 3 starting at 9 μM (**Figure 3E**). Moreover, Z-VAD-FMK, an irreversible pan-caspase inhibitor, was applied to block apoptosis. The results showed that Z-VAD-FMK increased cell viability and relieved drug-induced toxicity to HepG2 cells, as shown in **Figures 3F, G**.

**Figure 4:**
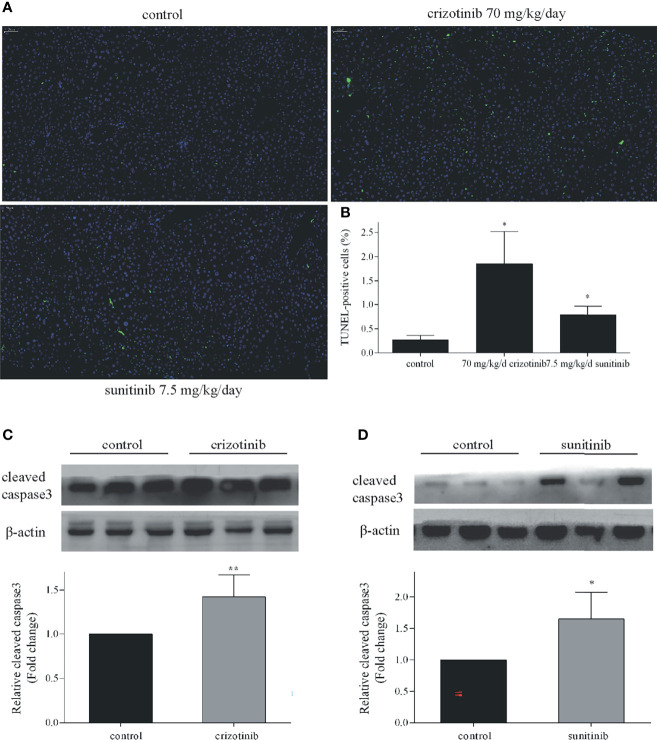
Crizotinib and sunitinib induced apoptosis *in vivo*. **(A)** The ICR mice hepatocyte apoptosis in liver tissue (TUNEL, 20×). The blue fluorescence indicates nuclei, and the green fluorescence indicates apoptotic cells. **(B)** TUNEL-positive cells were quantified. **(C, D)** The protein expression of cleaved caspase 3 in ICR mice treated with vehicle, crizotinib, or sunitinib treatment (n = 6). *P < 0.05 or **P < 0.01 (the crizotinib or sunitinib alone vs. control).

As shown in **Figures 4A, B**, the number of TUNEL-positive cells in the liver tissue of ICR mice increased significantly after crizotinib and sunitinib treatment. When crizotinib and sunitinib were applied to mice, the expression of cleaved caspase 3 was increased significantly, which was consistent with the results of TUNEL assay (**Figures 4C, D** **)**. These results further revealed that apoptosis and/or necrosis contributed to crizotinib- and sunitinib-induced hepatocyte death.

### Crizotinib and Sunitinib Induced Oxidative Stress

**Figure 5:**
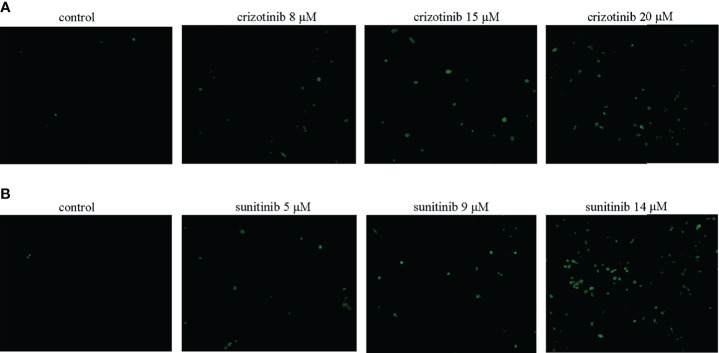
**(A, B)** Crizotinib and sunitinib increased ROS levels in HepG2 cells (n = 3). The intracellular ROS levels were measured using DCFH-DA. The microscopic images of the intensity of DCH fluorescence of respective experimental group (magnification ×200).

**Figure 6:**
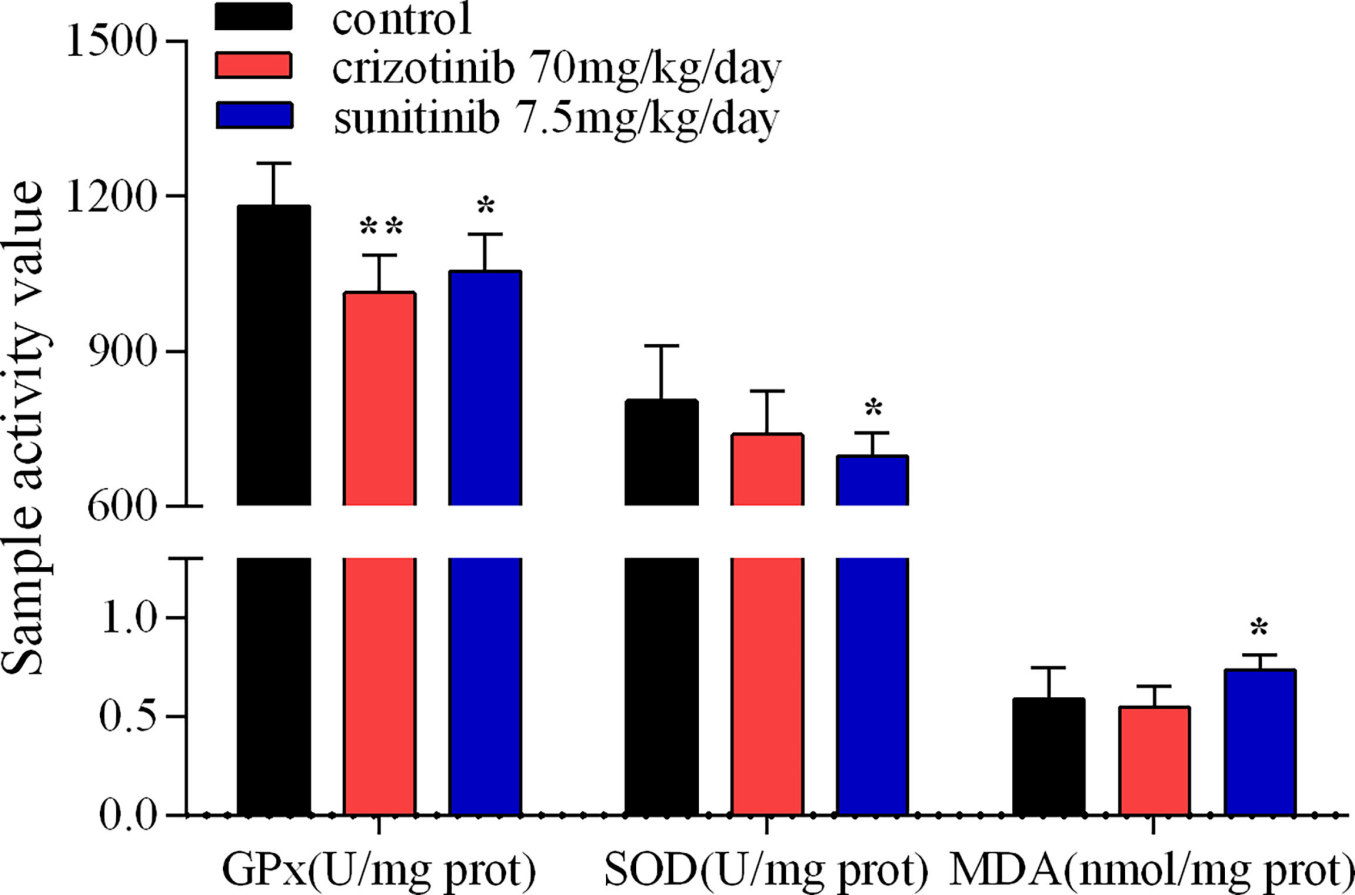
The activity assay of hepatic GPx, SOD and MDA in mice treated with crizotinib or sunitinib (n = 6-8). *P < 0.05 and **P < 0.01 (the crizotinib or sunitinib alone vs. control). GPx, glutathione peroxidase; SOD, superoxide dismutase; MDA, malondialdehyde.

**Figure 7:**
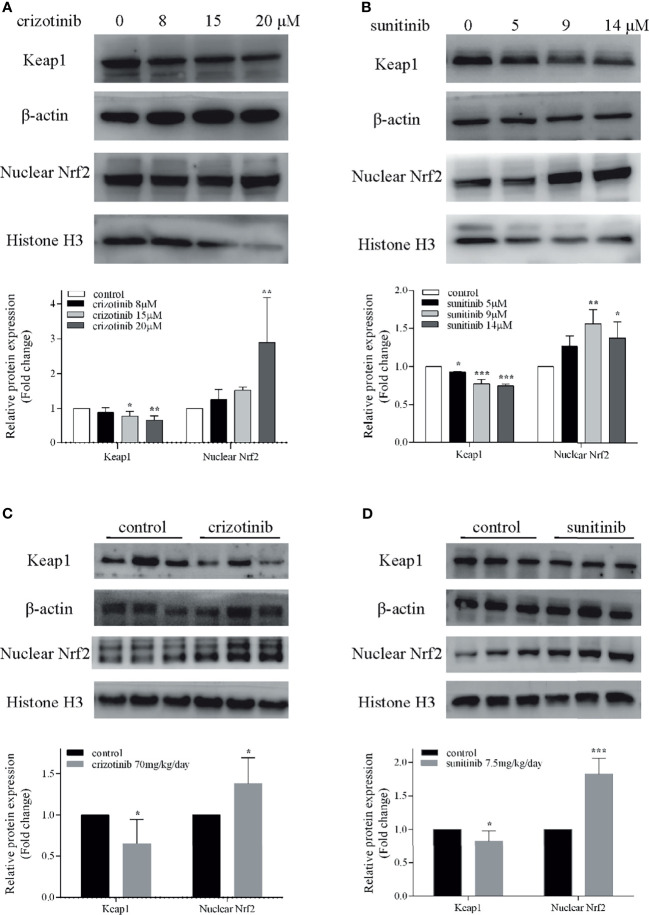
Involvement of the Nrf2 pathway in crizotinib- or sunitinib-mediated hepatotoxicity *in vitro* and *in vivo*. **(A, B)** The protein expression of Keap1 and nuclear Nrf2 in HepG2 cells (n = 3). **(C, D)** The hepatic Keap1 and nuclear Nrf2 protein levels in mice treated with crizotinib or sunitinib alone (n = 6). β-actin: loading control of total protein; Histone H3: nuclear loading control. *P < 0.05, **P < 0.01 or ***P < 0.001 (the crizotinib or sunitinib alone vs. control). Keap1, Kelch-like ECH-associated protein-1; Nrf2, Nuclear factor erythroid-2 related factor 2.

As shown in **Figures 5A, B**, treatment with crizotinib or sunitinib (24 h) increased the production of ROS in a concentration-dependent manner compared with control cells. As shown in **Figure 6**, a significant reduction in the activity of GPx was found in both crizotinib- and sunitinib-treated animals compared to the control group. However, accumulation of MDA and a decrease of the activity of SOD were significantly observed in the sunitinib but not crizotinib treatment group. Accordingly, The function of the endogenous antioxidant defense system is impaired as demonstrated by a decrease of SOD activity and an increase of MDA which cannot remove ROS effectively leading to the accumulation of ROS in the liver tissues of mice. Subsequently, we investigated the changes in the Keap1/Nrf2 pathway which played an important role in oxidative stress. When HepG2 cells were exposed to crizotinib or sunitinib for 24 h, the protein expression of total Keap1 was down-regulated while nuclear Nrf2 was up-regulated (**Figures 7A, B** **)**. Similar to *in vitro* findings, crizotinib- and sunitinib-treated animal groups showed down-regulation and up-regulation for the expression of Keap1and nuclear Nrf2, respectively (**Figures 7C, D** **)**.

### The Mitochondrial Dysfunction Was Involved in Crizotinib- and Sunitinib-Induced Hepatotoxicity

**Figure 8:**
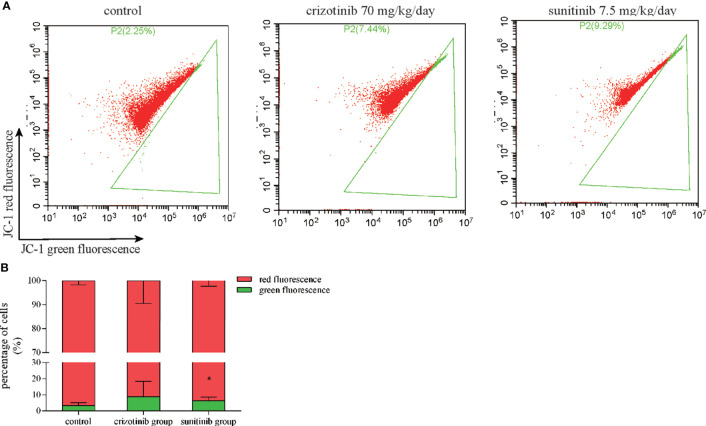
Crizotinib and sunitinib disrupt MMP in liver tissue. **(A)** Treatment with crizotinib or sunitinib decreased MMP in animals treated for 4 weeks compared to the control group as measured by flow cytometry and JC-1 staining. Red fluorescence represents JC-1 aggregates in the normal mitochondria whereas green fluorescence represents JC-1 monomer indicating MMP dissipation. **(B)** Quantification of high- and low-MMP cells in liver tissue (n = 8). *P < 0.05 (the crizotinib or sunitinib alone vs. control). MMP, mitochondrial membrane potential.

**Figure 9:**
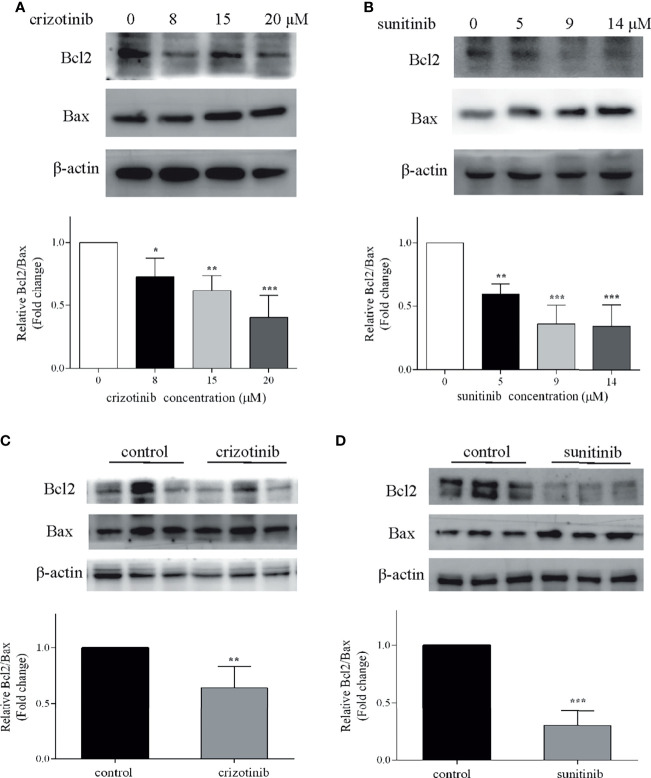
Crizotinib or sunitinib treatment altered the levels of Bcl2 and Bax in HepG2 cells and in liver tissue. **(A, B)** Protein levels of Bcl2 and Bax in HepG2 cells treated with crizotinib or sunitinib for 24 h (n = 3). **(C, D)** Protein level of Bcl2 and Bax in liver tissue in mice treated with crizotinib or sunitinib for 4 weeks (n = 6). *P < 0.05, **P < 0.01 or ***P < 0.001 (the crizotinib or sunitinib alone vs. control).

Mitochondria are a crucial component of the intrinsic pathway of apoptosis, a major mechanism of drug-induced cytotoxicity. MMP is an important indicator of mitochondrial function. In **Figure 8**, red fluorescence represents JC-1 aggregates in the normal mitochondria whereas green fluorescence represents JC-1 monomer indicating MMP dissipation. When the ratio of red-to-green fluorescence intensity decreases, it indicates a loss of MMP that is widely probed by JC-1 staining. *In vivo*, flow cytometry results showed that the ratio of JC-1 aggregates/JC-1-monomer was reduced in the crizotinib- and sunitinib-treated groups, indicating the impairments of MMP (**Figures 8A, B** **)**. Also, crizotinib and sunitinib altered the balance between the anti-apoptotic protein Bcl2 and the pro-apoptotic protein Bax on the mitochondrial membrane **(** **Figures 9A, B** **)**. *In vivo*, compared with the untreated group, crizotinib and sunitinib induced a concentration-dependent decrease in the Bcl2/Bax ratio **(** **Figures 9C, D** **)**.

## Discussion

Small molecule kinase inhibitors, such as tyrosine kinases inhibitors (TKIs), which are designed to inhibit the action of mutated or over-expressed tyrosine kinases in cancer cells, have improved the management of cancers and significantly extended survival in cancer patients compared with traditional chemotherapy agents (23). However, unexpected toxic reaction of hepatotoxicity has been reported for several TKIs, including imatinib, gefitinib, sunitinib, crizotinib, lapatinib, pazopanib, ponatinib, and regorafenib (11, 24, 25). As of October 2019, the FDA has approved 53 small molecule kinase inhibitors, seven (sunitinib, lapatinib, pazopanib, regorafenib, ponatinib, idelalisib, pexidartinib) of which had a black box warning of liver toxicity, and twenty-nine of which had warnings and precautions for hepatotoxicity in their product labeling (26). Many case reports demonstrated that crizotinib and sunitinib induced hepatotoxicity, even acute liver failure (ALF) (27–29). However, dose adjustment or drug discontinuation are the common strategies to reduce or manage hepatotoxicity induced by crizotinib or sunitinib. Also, alternative agents such as alectinib though belongs to the same drug class, could be a choice in cases of crizotinib-induced liver toxicity, however more evidence is awaited (28). Thus, monitoring of liver function is recommended for patients using crizotinib or sunitinib, especially in patients with liver impairment or those using antisecretory drugs (30). Furthermore, applying the above-described measures may contribute to treatment failure and tumor progression in some cases. A limited number of systematic studies described the molecular mechanism(s) associated with crizotinib- and sunitinib-induced hepatotoxicity. Therefore, it is necessary to elucidate the molecular mechanisms and pathways associated with crizotinib- and sunitinib-induced liver toxicity.

In this study, we established an animal model that mimicked the clinical dose and duration of administration of crizotinib and sunitinib to investigate their hepatotoxicity. In addition, HepG2 cells are a well-characterized human cell system suitable for investigating mitochondrial drug toxicity (31, 32). Findings from our study demonstrated that crizotinib and sunitinib treatment reduced viability of HepG2 cells and induced liver toxicity in animal model. A study indicated that the pattern of liver injury in patients receiving TKIs is typically hepatocellular (29), so we investigated the main way of hepatocyte death caused by crizotinib and sunitinib. Apoptosis and necrosis are the two major forms of cell death, which are relevant to drug-induced liver injury (33, 34). In our study, the flow cytometry results demonstrated that the percentage of HepG2 cells undergoing apoptosis or necrosis is increased in crizotinib-treated cells when compared with the untreated hepatocytes, consistent with previously published reports (14, 15, 35). Although sunitinib cannot be treated with fluorescent dyes to investigate apoptosis because of autofluorescence, Western blotting demonstrated that the level of cleaved caspase 3 increased in HepG2 cells and liver tissue after both crizotinib and sunitinib treatment. Meanwhile, the results of crizotinib- and sunitinib-mediated apoptosis were also confirmed by TUNEL assay *in vivo*. In addition, Z-VAD-FMK, the caspase inhibitor, effectively protected from drug-induced liver cell death and reduced the release hepatic enzymes ALT, AST, and LDH caused by crizotinib and sunitinib.

Mitochondria play an important role in oxidative stress and the intrinsic apoptotic pathway (36). Bcl2 and Bax proteins are important regulators factors of MMP. Bcl2/Bax ratio can control the release of cytochrome C from mitochondria and the activation of downstream caspase 3 to promote cell survival or apoptosis (37, 38). Previous studies indicated that crizotinib dissipated MMP starting at high concentrations (starting at 50 μM) and inhibited glycolysis only weakly when applied to HepG2 cells (14), and MMP was not affected in rat liver mitochondria (13). Notably, we found that crizotinib could dissipate the MMP by decreasing the expression of Bcl2/Bax in the liver tissue. In addition, an *in vitro* study reported that sunitinib has mitochondrial toxicity, which reduced the MMP starting at 1 μM in HepG2 cells and after exposure for 15 min at 10 μM in isolated mouse liver mitochondria (19). However, there were other reports that sunitinib did not disrupt the MMP of rat heart mitochondria (39), mouse liver mitochondria (40), and isolated rat liver mitochondria (13). In our study, after sunitinib treatment, the MMP of liver tissue dissipated significantly and the expression of Bcl2/Bax decreased significantly. The different findings can be explained by differences in the experimental models and settings applied according to Peach et al. (17). Taken together, our findings demonstrate regulatory roles for Bcl2 and Bax in altering MMP in crizotinib- and sunitinib-induced mitochondrial apoptotic pathway.

In a case report by Kreitman et al., treatment with N-acetylcysteine (NAC), a ROS scavenger, partially restored liver function tests to normal level and partially relieved ALF induced by crizotinib in a patient (27). In line with this, we previously indicated that NAC treatment decreased hepatocyte damage induced by crizotinib and sunitinib in HL7702 cells (35). Therefore, the results of these efforts indicated that the underlying mechanism might be related to oxidative stress. Oxidative stress results from an imbalance between ROS and antioxidants, which has long been recognized as a critical pathogenic factor in acute injury, including acute kidney injury and acute liver injury (41, 42). The overproduction of ROS can reduce the content of GPx and SOD which are two major antioxidant enzymes to reduce oxidative stress. Meanwhile, high levels of ROS can cause lipid peroxidation to damage cellular membranes, and MDA is a significant marker of lipid peroxidation (43). Our research revealed that crizotinib and sunitinib significantly increased the level of ROS in a concentration-dependent manner in HepG2 cells, and markedly reduced the content of GPx and SOD, and increased MDA in liver tissue. However, the change of ROS was not statistically significant in experimental animals. Possible reasons for the variability in results might include differences in animal models used, the drug dose used, and the experimental assays used to detect ROS in isolated liver mitochondria. These results demonstrate that the imbalance between ROS and antioxidative function leads to oxidative stress, which contributes to hepatocyte damage.

Currently, strategies for the prevention and treatment of hepatotoxicity induced by TKIs are very limited, and it is necessary to find the key targets in TKIs-induced liver injury. Nrf2, which is an imperative redox-sensitive transcription factor targeting of elimination of ROS, and its activation is widely thought to alleviate the liver diseases triggered by oxidatie stress (44, 45). Under stress conditions, Nrf2 dissociates from Keap1, translocates to the nucleus and binds to antioxidant response elements, which results in the expression of diverse antioxidant and metabolic genes to relieve oxidative stress (46, 47). Importantly, we first found that low doses of crizotinib and sunitinib activated the Keap1/Nrf2 signaling pathway *in vitro* and *in vivo* to alleviate self-induced hepatotoxicity, which is following previously published papers on drug-induced liver injury (42, 48, 49). Therefore, our findings indicated that the activation of the Keap1/Nrf2 signaling pathway could be a potential therapeutic target for TKIs in the treatment of liver injury.

## Conclusions

The results show that crizotinib and sunitinib induce hepatic oxidative stress and apoptosis that lead to hepatotoxicity. The activation of the Keap1/Nrf2 signaling pathway was involved in crizotinib- and sunitinib-induced oxidative stress, which might partially protect against their induced oxidative damage. However, the specific mechanism underlying the relationship between crizotinib- and sunitinib-induced oxidative stress and mitochondrial apoptotic pathway requires further investigations. Therefore, we will continue to explore additional biomarkers for hepatotoxicity and other potential signaling pathways associated with crizotinib- and sunitinib-induced liver injury.

## Data Availability Statement

The original contributions presented in the study are included in the article/supplementary material. Further inquiries can be directed to the corresponding authors.

## Ethics Statement

The animal study was reviewed and approved by the Institutional Animal Care and Use Committee of Central South University (Hunan, China).

## Author Contributions

LG contributed to conceiving and designing the experiments. TT and DF performed the data analyses and wrote the manuscript. LZ and MY contributed significantly to analysis and manuscript preparation. HG, BZ, and YZ helped perform the analysis with constructive discussions. All authors contributed to the article and approved the submitted version.

## Funding

This research was funded by the National Natural Science Foundation of China, grant number 81974532 and No. 81803830, the Natural Science Foundation of Hunan Province, China, grant number 2020JJ4130, and Science and Technology Department of Hunan Province, China, grant number 2017SK1030.

## Conflict of Interest

The authors declare that the research was conducted in the absence of any commercial or financial relationships that could be construed as a potential conflict of interest.

## Publisher’s Note

All claims expressed in this article are solely those of the authors and do not necessarily represent those of their affiliated organizations, or those of the publisher, the editors and the reviewers. Any product that may be evaluated in this article, or claim that may be made by its manufacturer, is not guaranteed or endorsed by the publisher.
